# A Novel Surgical Technique for Fixation of Recurrent Acromioclavicular Dislocations: AC Dog Bone Technique in Combination with Autogenous Semitendinosus Tendon Graft

**DOI:** 10.1155/2017/5457625

**Published:** 2017-05-23

**Authors:** Patrick Holweg, Wolfgang Pichler, Gerald Gruber, Ellen Tackner, Franz Josef Seibert, Patrick Sadoghi, Gloria Hohenberger

**Affiliations:** ^1^Department of Trauma Surgery, Medical University of Graz, Graz, Austria; ^2^Department of Orthopaedic Surgery, Medical University of Graz, Graz, Austria

## Abstract

Various surgical techniques have been described for the fixation of acromioclavicular (AC) dislocations. However, recurrent dislocation is one of the main complications associated with the majority of these techniques. We report a case of postoperative AC joint redislocation. In order to overcome recurrent dislocation after revision surgery, a reconstruction of the conoid and trapezoid ligament with the use of a free tendon graft in combination with a FiberTape was provided within a novel surgical technique. After 12 months, the patient was very satisfied with the functional outcome. The patient achieved excellent results in the Constant (98 points), SPADI (0 points), and QuickDASH score (0 points). The described technique results in an anatomic reconstruction of the AC joint. The nonrigid nature of the intervention seems to restore the normal arthrokinematics by reconstructing the coracoclavicular ligaments with an autograft which is then protected by the AC Dog Bone artificial ligaments during the healing period. The arthroscopic approach to the AC joint with minimal exposure reduces the risks and complications of the intervention. This is the first case in literature that utilizes the artificial dog bone ligament securing the autograft in an anatomic AC reconstruction.

## 1. Introduction

Various surgical techniques have been described for the fixation of acromioclavicular (AC) dislocations [1]. However, recurrent dislocation is one of the major complications associated with most of these techniques.

In order to overcome these, a reconstruction of the conoid and trapezoid ligaments with the use of a free tendon graft seems to provide the most durable construct [2]. We hereby present a novel technique by combining the AC Dog Bone Fibertape (Arthrex, Inc., Naples, Florida) with an autogenous semitendinosus tendon graft, in order to achieve maximum primary stability as well as using a free tendon graft to biologically reconstruct the ligaments.

## 2. Patient Information 

**Figure 1:**
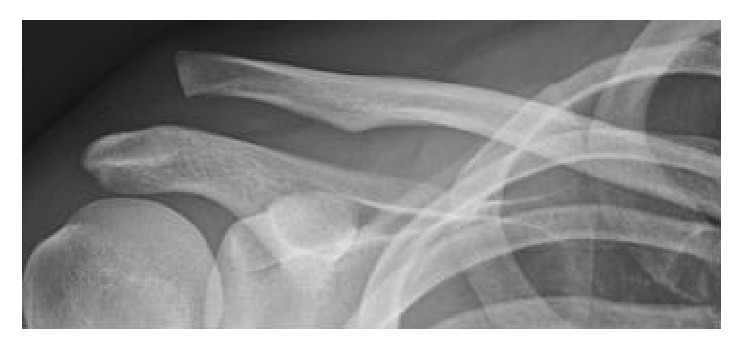
Radiograph of a 24-year-old male patient after a cycling accident; dislocation of the AC joint (Rockwood V).

A 24-year-old male patient involved in a cycling accident sustained an acute AC joint dislocation of Rockwood type V [3] (Figure 1).

**Figure 2:**
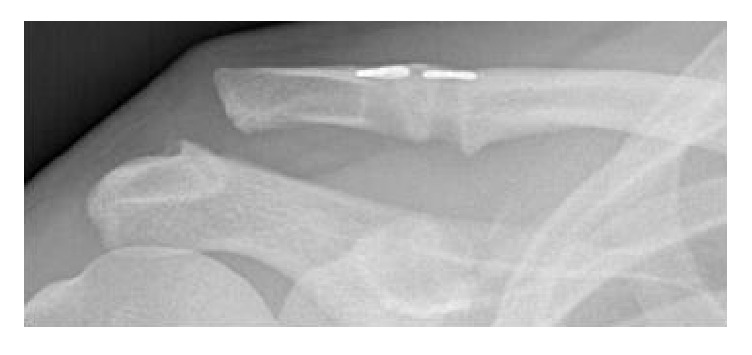
Recurrent trauma eight months after surgery; redislocation of the AC joint.

He was treated surgically one week after the injury. Two TightRopes (Arthrex, Inc., Naples, Florida) were inserted arthroscopically to reduce the AC joint anatomically. The patient went to have a full and uneventful recovery. Eight months later, he got involved in a further cycling accident and reinjured his shoulder. Therefore, radiographs showed a redislocation of the AC joint (Figure 2). Revision surgery was decided since the patient had high athletic demands.

## 3. Surgical Treatment

We used the AC Dog Bone Technique in combination with an autogenous semitendinosus tendon graft aiming to achieve a maximum primary stability in combination with a tendon graft as biological ligament replacement.

Under general anesthesia the patient was positioned in a beach chair position. Standard dorsal, anterior inferior, and lateral arthroscopic portals were used. After exposition of the coracoid, the TightRope buttons and the fiberwires were exposed and removed.

**Figure 3:**
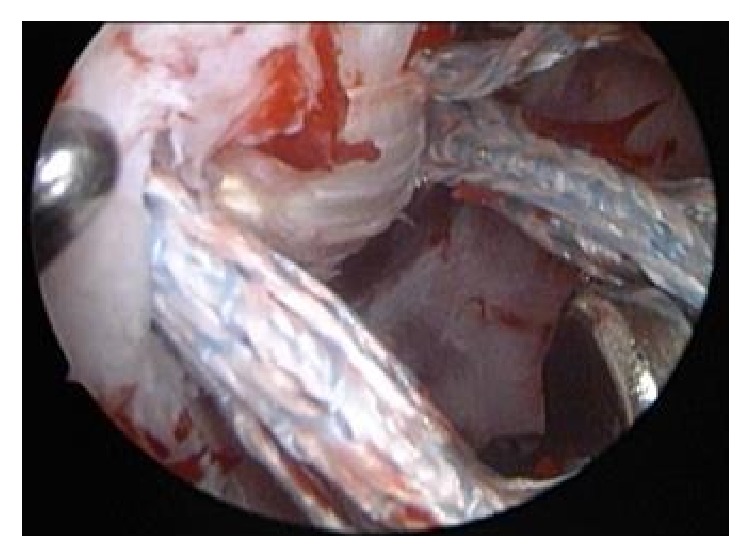
Arthroscopic view of the coracoid base; the semitendinosus graft passes through the holes at the coracoid base and is already tensioned; the FiberTapes are still loose.

**Figure 4:**
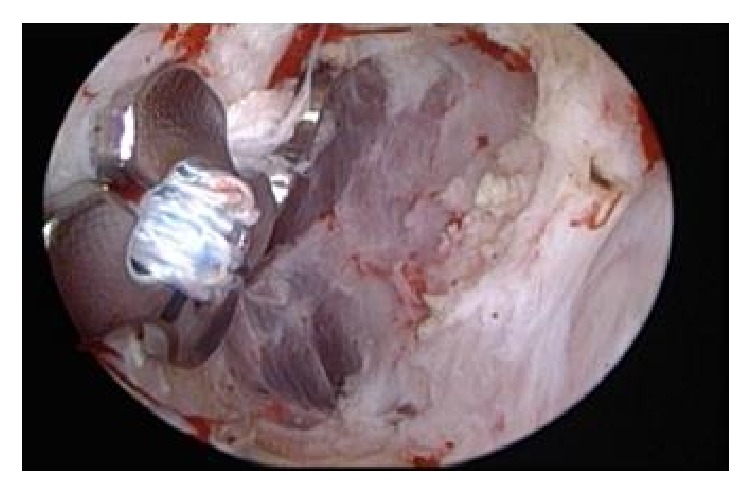
Arthroscopic view of the coracoid base; the dog bone buttons are finally tied to the coracoid base above the semitendinosus graft.

**Figure 5:**
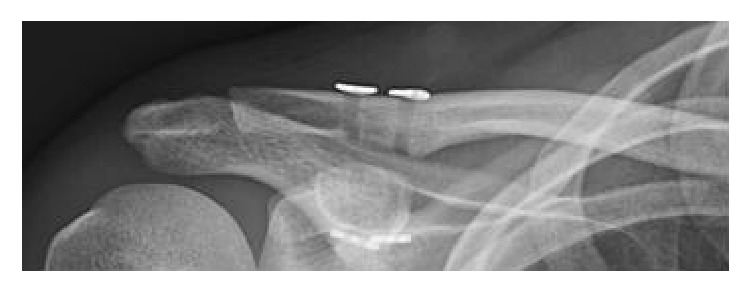
Postoperative radiograph with anatomic reduction of the AC joint; two visible drilling holes and four dog bone plates.

Guide wires were positioned through the preexisting drill holes in the clavicle and coracoid. All holes were drilled again by using the TightRope 4 mm drill. SutureLasso Wire Loops (Arthrex, Inc., Naples, Florida) were passed through the drill holes. FiberTapes were loaded through the SutureLasso Wire Loop and pulled up through the coracoid and clavicle tunnels. SutureLasso was again passed through the clavicle and coracoid tunnels. The semitendinosus tendon was harvested from the ipsilateral knee in a standard manner. The tendon was attached to the Wire Loop and pulled through the tunnels. This is illustrated in Figure 3. Anatomical reduction of the AC joint was confirmed intraoperatively with an image intensifier and a K-wire (1.8 mm) was used to temporarily hold the reduction. FiberTapes and tendon graft were tensioned alternately, as shown in Figures 3 and 4. The tendon was fixed to the clavicle with a 3.5 mm bioabsorbable screw. Dog bone plates were inserted and FibreTapes were tied and the K-wire removed. Since the patient had developed posttraumatic AC arthritis, he required an additional excision of the lateral border of the clavicle (Figure 5).

## 4. Follow-Up and Outcomes

**Figure 6:**
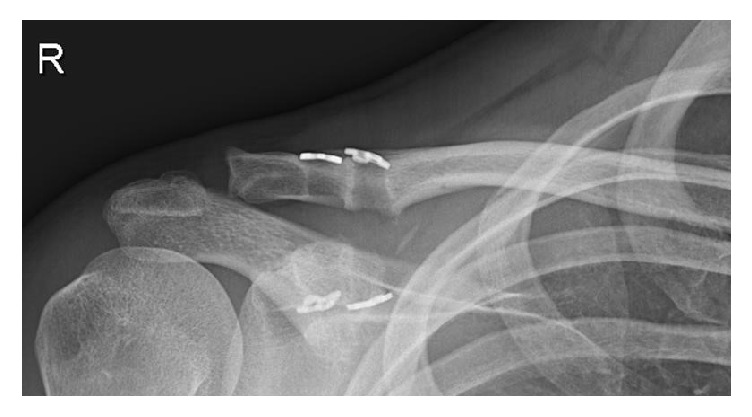
Radiograph 12 months after intervention with anatomic reduction of the AC joint; two visible drilling holes and four dog bone plates.

The treated extremity was immobilised by use of a shoulder bandage for a durance of 6 weeks following surgery. Passive motion of the shoulder was started 3 weeks after the intervention and was advanced to exercises against resistance 6 weeks following surgery. Sportive activities were recommended to be avoided for 12 weeks. The patient was reevaluated after six weeks, four months, and 12 months. Besides the clinical evaluation, radiographs of the operated AC joint were taken (Figure 6). After 12 months the patient was very satisfied with the functional outcome. He presented with a free range of motion in the injured shoulder joint without any pain at rest or movement. The patient achieved excellent results in the Constant (98 points), SPADI (0 points), and QuickDASH score (0 points).

## 5. Discussion

In 1972 Weaver and Dunn first described the treatment of AC dislocations through excision of the lateral border of the clavicle and transfer of the coracoacromial ligament to the clavicle [4]. Since the transferred ligament is weaker than the native coracoclavicular ligaments, recurrence of the dislocation is a common complication. Numerous modifications of this operation technique have been reported in order to reduce the risk of secondary dislocations [5–7].

Currently there are four main surgical options for AC joint disruptions: (1) primary AC joint fixation (using pins, screws, suture wires, plates, and the hook plate) with or without ligament repair reconstruction; (2) primary fixation of the coracoclavicular interval with or without acromioclavicular ligament reconstruction; (3) distal clavicle excision with or without coracoclavicular ligament repair; (4) muscle transfer with or without distal clavicle excision [8].

To the authors' knowledge, there is no gold standard therapy option for the treatment of AC joint dislocations.

Each described technique has its limitations. The commonly used hook plate can lead to chronic irritations of the subacromial space resulting in a persisting pain syndrome [9]. For this reason, removal of the plate is recommended after six months, necessitating a further surgical procedure with associated possible complications. A report has shown that rigid fixation with the hook plate does not result in a decreased incidence of recurrent subluxation of the AC joint [9]. Recent efforts have focused on achieving a stable but nonrigid fixation to preserve normal arthrokinetics of the joint.

In their biomechanical study Tashjian et al. [10] demonstrated some displacement of the AC joint in a superior direction following reconstruction using a tendon graft without augmentation after cycling loadings. Other biomechanical studies reported that a coracoclavicular reconstruction using a free tendon graft like the AC graft rope system (Arthrex Inc.) provides almost equal stability as the native coracoclavicular ligament [2, 11].

Lädermann and colleagues [12] treated 37 patients who had sustained grade III-V AC joint dislocations via refixation with nonabsorbable sutures and received good outcomes including a mean DASH of 7. In Martetschläger et al. [13], 24 cadaveric shoulders underwent AC reconstruction with two coracoclavicular as well as one acromioclavicular PDS-cerclage. Here, biomechanical testing did not provide satisfactory vertical joint stability. Breslow and colleagues [14] compared AC refixation with suture anchors to its reconstruction with nonabsorbable sutures in six paired cadaveric shoulders. Authors observed an equal ability of stabilisation for both techniques.

Concerning further devices, Walz et al. [15] proved the fixation with two TightRope devices in a cadaveric model to be a stable technique. Furthermore, Rosslenbroich and colleagues [16] observed a good clinical outcome regarding 96 patients who had undergone AC reconstruction with a fixation button (Fliptack).

The technique reported hereby does not require an open approach to the joint which minimizes the risks and complications. Using a free tendon graft in combination with the AC Dog Bone FiberTape Technique (Arthrex, Inc., Naples, Florida) combines the advantages of both methods. The free tendon graft provides an anatomic reconstruction of the ligament with biological properties, whereas the AC Dog Bone 2 System secures the anatomic reduction in the early stage of tendon graft healing. This novel surgery is appropriate for acute and chronic AC disruptions. A high primary stability is obligatory for both injury patterns, which is ensured by the artificial ligaments. Especially in chronic cases, it is very important to substitute the injured ligaments by autogenous ligaments. This is achieved by implanting a semitendinosus autograft. This is the first case in literature that utilizes the artificial dog bone ligament securing the autograft in an anatomic AC reconstruction.

## 6. Conclusion

The hereby described technique results in an anatomic reconstruction of the AC joint. The nonrigid nature of the technique restores the normal arthrokinematics by reconstructing the coracoclavicular ligaments with an autograft which is then protected by the AC Dog Bone artificial ligaments until full incorporation. The arthroscopic approach to the joint with minimal exposure reduces the risks and complications of the operation.

Removal of the AC Dog Bone implant is not necessary due to its low profile. This technique is a novel, anatomical, and nonrigid reconstruction of the AC joint, aiming for the advantages and minimizing the limitations of previously described techniques.
